# The Impact of Li Grain Size on Coulombic Efficiency in Li Batteries

**DOI:** 10.1038/srep34267

**Published:** 2016-10-05

**Authors:** B. Layla Mehdi, Andrew Stevens, Jiangfeng Qian, Chiwoo Park, Wu Xu, Wesley A. Henderson, Ji-Guang Zhang, Karl T. Mueller, Nigel D. Browning

**Affiliations:** 1 Joint Center for Energy Storage Research (JCESR), Pacific Northwest National Laboratory (PNNL), Richland, WA 99352, USA; 2 Physical and Computational Science Directorate, PNNL, Richland, WA 99352, USA; 3 National Security Directorate, PNNL, Richland, WA 99352, USA; 4 Energy and Environmental Directorate, PNNL, Richland, WA 99352, USA; 5 Industrial and Manufacturing Engineering, Florida State University, Tallahassee, FL 32306, USA; 6 Environmental Molecular Sciences Laboratory, PNNL, Richland, WA 99352, USA; 7 Department of Chemistry, Penn State University, University Park, PA, 16802, USA; 8 Materials Science and Engineering, University of Washington, Seattle, WA 98195, USA

## Abstract

One of the most promising means to increase the energy density of state-of-the-art lithium Li-ion batteries is to replace the graphite anode with a Li metal anode. While the direct use of Li metal may be highly advantageous, at present its practical application is limited by issues related to dendrite growth and low Coulombic efficiency, CE. Here *operando* electrochemical scanning transmission electron microscopy (STEM) is used to directly image the deposition/stripping of Li at the anode-electrolyte interface in a Li-based battery. A non-aqueous electrolyte containing small amounts of H_2_O as an additive results in remarkably different deposition/stripping properties as compared to the “dry” electrolyte when operated under identical electrochemical conditions. The electrolyte with the additive deposits more Li during the first cycle, with the grain sizes of the Li deposits being significantly larger and more variable. The stripping of the Li upon discharge is also more complete, i.e., there is a higher cycling CE. This suggests that larger grain sizes are indicative of better performance by leading to more uniform Li deposition and an overall decrease in the formation of Li dendrites and side reactions with electrolyte components, thus potentially paving the way for the direct use of Li metal in battery technologies.

Over the years there have been many attempts to eliminate the formation of dendrites in Li metal batteries using various means including by increasing the uniformity of the solid electrolyte interphase (SEI) layer^1^,^2^,^3^,^4^,^5^,^6^,^7^,^8^,^9^, alloying the Li metal deposits with other metals^10^,^11^, or using a polymer electrolyte to mechanically inhibit the growth^12^. Recently, it was demonstrated that dendrite formation was reduced and uniform Li deposition was achieved by using a highly concentrated electrolyte composed of 4M lithium bis(fluorosulfonyl)imide (LiFSI) in 1,2-dimethoxyethane (DME)^3^. This approach enabled high-rate cycling with a >99% Coulombic efficiency (CE) for the Li deposition/stripping without dendrite growth. A similar increase in the uniformity of the Li films grown on the anode during the charging process has been obtained by incorporating a CsPF_6_ additive into the electrolyte, which resulted in the formation of high density, self-aligned nanorod structures surrounded by the SEI layer^13^. Notably, LiPF_6_-based electrolytes containing trace amount of H_2_O (25–50 ppm) also suppress Li dendrite growth and lead to the same formation of Li columnar structures as noted for the CsPF_6_ additive (in contrast to the Li morphology obtained using an electrolyte containing only the residual H_2_O (~10 ppm) content), thus demonstrating that minor changes to the electrolyte’s chemical composition can completely transform the Li deposition morphology^14^.

To investigate how the morphology of the Li deposits leads to improved CE, we have employed an *operando* electrochemical cell (*ec*-cell), illustrated in Fig. 1, in an aberration-corrected scanning transmission electron microscope (STEM) to study the cycling process at high spatial resolution. This approach enables the direct observation of the initial stages of Li deposition/stripping in an electrolyte (1M LiPF_6_ in propylene carbonate (PC)) with controlled trace-amounts of H_2_O additive (10 and 50 ppm). In recent years, rapid growth has occurred in the use of *in-situ* liquid stages for TEM^15^ that, in addition to electrochemistry^16^,^17^,^18^,^19^,^20^,^21^, have enabled nucleation/growth phenomena^22^,^23^,^24^,^25^ and biological systems^26^ to be observed first-hand providing tremendous insight into molecular-level interactions. By using this stage to evaluate the initial stages of Li deposition, we have correlated the deviations in the microstructure evolution directly with the presence of the additive.

Figure 2 shows a summary of the key parts of an electrochemical deposition/stripping cycle for two electrolytes prepared and analyzed under identical conditions. During the CV cycling, the deposition and stripping of Li metal can be clearly delineated — i.e., since Li is less dense than the surrounding electrolyte, it can be identified by its “reversed” contrast in the STEM images^27^. As seen from the video captures at the start of the scan, at the end of the deposition and stripping processes there are significant differences in both the amount and morphology of the Li deposited and stripped using the two electrolytes (Fig. 2B–G). The amount of Li at the electrode-electrolyte interface can be quantified by using a multi-particle tracking algorithm^28^. In each of the video frames, the Li grains are not moving, but are instead actively nucleating and growing from the electrode surface into the electrolyte solution. To determine the changes in size and shape, we first applied an image thresholding method to each of the video frames to extract the outlines and the interiors of select Li nanoparticles/grains. We then combined this method with the M 1⁄4 3, Henriques’ method, Jaqaman’s method and MCMC Data Association to relate the individual extractions from the different time frames to track the nanoparticles and their interactions^28^. Figure 3A shows that the electrolyte with 50 ppm of the H_2_O additive exhibits a more rapid Li deposition and an increase in the amount of Li deposited at the peak of the deposition. More importantly, at the end of the stripping process, there is less overall Li remaining at the interface. This means that the electrochemical process in the electrolyte with the additive is more reversible than in the one without it, i.e., it has a higher CE (85% compared to 75% for the electrolyte without the additive). We can also use the tracking algorithm to measure the grain size of the Li deposits (Fig. 3B). There are issues with the overlap of grains (the image is a 2-D projection of an evolving 3-D structure) making it difficult to measure all of the grains independently. For both of the experiments, however, there are 8 grains that can be clearly identified throughout the CV scans (Figures S2 and S3). In the case of the electrolyte without the additive, the grain size is small and relatively uniform with clearly defined interfaces (grain boundaries). In contrast, for the electrolyte with the additive, the grain sizes are 2–3 times larger on average with a much wider variability in size. Thus, there are fewer grain boundaries in the deposits that form with the additive. As we will discuss later in this paper, the grain size of the electrodeposit appears to correlate directly with CE.

To understand the origins of the differences in the morphology of the electrodeposited Li in the two electrolytes, the chemistry that is occurring in the presence of an increased H_2_O content (50 ppm) must be considered. When H_2_O is added to the electrolyte, it reacts as follows:
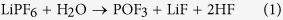


The electrolyte with the additive therefore has an increased HF content relative to the one without the additive. This, in turn, promotes the formation of LiF:
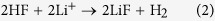


LiF is a well-known component within the solid electrolyte interphase (SEI) layer that forms during the cycling of Li-ion batteries^1^,^2^, which, during electrodeposition, has been shown to be highly effective at increasing the ionic conductivity of the system. Other well-known components of the SEI layer include lithium alkyl carbonates (e.g., C_3_H_6_(OCO_2_Li)), LiCO_3_, and Li_2_O, in addition to other organic products from the solvent. Note that, for the initial stages of the Li deposition/stripping in the Li battery shown in Fig. 1, the term SEI layer refers to the electrochemical products formed at the interface.

Each of the SEI components has a different contribution to the ionic and electronic conductivities of the interface and LiF, in particular, has much higher conductivities than the other components. It is this which we believe is the origin of the dramatic effect on the Li plating morphology and its relationship to the cycling characteristics (the additive is present at the ppm level, meaning that it is widely distributed and will only affect certain areas during the electrodeposition process). Recent work on the failure mechanisms which manifest under fast-charging conditions has shown that the SEI layer consists of highly porous regions of organic electrolyte products^29^. Fast-charging increases the concentration of the non-conducting porous/organic regions relative to the amount of the Li metal deposited, resulting in tortuous conducting paths and an increase in the overall battery impedance due to the formation of a non-uniform layer with poor electronic/ionic conductivity. For the electrolyte with 50 ppm of H_2_O, the increased amount of LiF formed increases the ionic conductivity of the SEI layer, which in turn increases the round trip efficiency of the Li deposition/stripping processes, resulting in a lower impedance and decreased buildup of “dead Li” (more of the electron density at the interface reacts with the continuous supply of Li^+^ cations rather than neighboring electrolyte components), as compared to the electrolyte with only 10 ppm of H_2_O. Similarly, the higher conductivity facilitates larger grain growth and the subsequent stripping of the Li metal as the grains remain in electrical contact with the electrode. The result is a more uniform Li and SEI layer deposition, an absence of dendritic Li growth and a reduction in the accumulation of electrolyte degradation products and dead Li metal (sometimes called mossy Li)^13^.

The effect of the SEI layer conductivity on the morphology of the electrodeposits was further validated by studying galvanostatic charging under constant current conditions of 1 and 4 mA cm^−2^; this permits direct monitoring of the rate of Li deposition/stripping for a set time period. Figure 4A shows the current density of the cell as a function of time for 1 mA cm^−2^ (the data for 4 mA cm^−2^ are reported in Figure S4). Figure 4B shows BF (left) and HAADF (right) frames from the movies (Movies SM3 and SM4) in which we focus primarily on the insertion and accumulation of Li into the Pt electrode^27^. As seen from Fig. 4C (using the tracking algorithms to measure the electrode size from the Movies SM3-SM6), the effect of the additive promotes faster Li^+^ cation diffusion and larger Li grain growth at the electrode. Under fast-charging conditions (4 mA cm^−2^), the Pt electrode itself degrades for the electrolyte without the additive. In stark contrast, the electrolyte with the additive is stable under the fast-charging conditions suggesting that the diffusion of Li^+^ cations through the SEI layer is faster with the additive, i.e., under the fast-charging conditions, the electrolyte without the additive cannot supply enough Li^+^ cations to the electrode surface, resulting in electrostatic breakdown. These results again suggest that larger grain sizes of the electrodeposits are desirable for better CE.

The quantified observations shown in Figs 3 and 4 allow us to understand the impact of grain morphology on the deposition/stripping processes and the CE shown in Fig. 5. The formation of LiF in the SEI layer provides “fast” Li^+^ cation diffusion channels that lead to the rapid deposition of Li on the surface of the electrode (and during discharge, the rapid stripping of Li and thus the higher cycling CE). Extended over many cycles, the increase in the CE and ease of the Li deposition/stripping will result in a much more compact Li layer on the surface, a reduction in the formation of dendrites and the minimization of SEI and dead Li buildup on the electrode surface.

## Conclusions

In conclusion, we have demonstrated that the formation of Li dendrites can be effectively controlled and suppressed by understanding and optimizing the effect of the H_2_O content on the morphology of the Li grains. The elevated H_2_O content increases the total concentration of HF in the electrolyte and consequently increases the LiF concentration in the SEI layer, which leads to the abnormally large grain growth of Li as opposed to the “mossy” Li formed in the dry electrolyte. The appearance of large grains of Li in the electrodeposit can be taken as a direct measurement of the improved CE of the battery — large grains are more desirable for high CE values, while small randomly interconnected grains are not. The LiF-rich SEI layer is able to sustain faster Li^+^ cation diffusion across the electrolyte-electrode interface through the highly conductive LiF channels, in stark contrast to what occurs for the lower concentration of LiF. Such analyses indicate that controlling the diffusion of Li^+^ cations through the SEI layer defines the Li grain size and presents a critical opportunity to tailor the performance of Li metal anodes, which may lead to their successful incorporation into next generation batteries.

## Methods

### Materials

The electrolyte-grade lithium hexafluorophosphate, LiPF_6_, and propylene carbonate, PC, were purchased from BASF and used without further purification. The specific H_2_O content in the electrolytes was determined by Karl Fischer titration, while the HF level was estimated using an acid–base titration technique^30^. The water content in the PC solvent and 1M LiPF_6_ in PC electrolyte were 13 and 10 ppm H_2_O, respectively. The additional amount of H_2_O (raising the amount to 50 ppm) was introduced after preparing the 1M LiPF_6_ in PC electrolyte using a micropipette. The precise water content in the electrolyte during the hydrolysis reaction was measured at specific time intervals by monitoring the amount of the H_2_O and HF in the electrolytes.

### Characterization

The *in-situ ec*-(S)TEM experiments shown here were performed on a FEI 80–300 kV Cs corrected Titan microscope equipped with a Schottky field-emission electron source and a CEOS hexapole spherical aberration probe corrector. For these experiments, the microscope was operated at 300 kV in both bright field (BF) and high angle annular dark field (HAADF) modes. All images were obtained after calibration of the electron dose, which was kept below ≤0.3 electrons Å^−2^ s^−1^ to avoid beam damage effects^18^,^28^. Under these conditions, typical beam effects such as the formation of bubbles and/or damage resulting in the formation of precipitates from the breakdown of the electrolyte^18^ are completely avoided.

### *In-situ* electrochemical measurements

**Figure 1:**
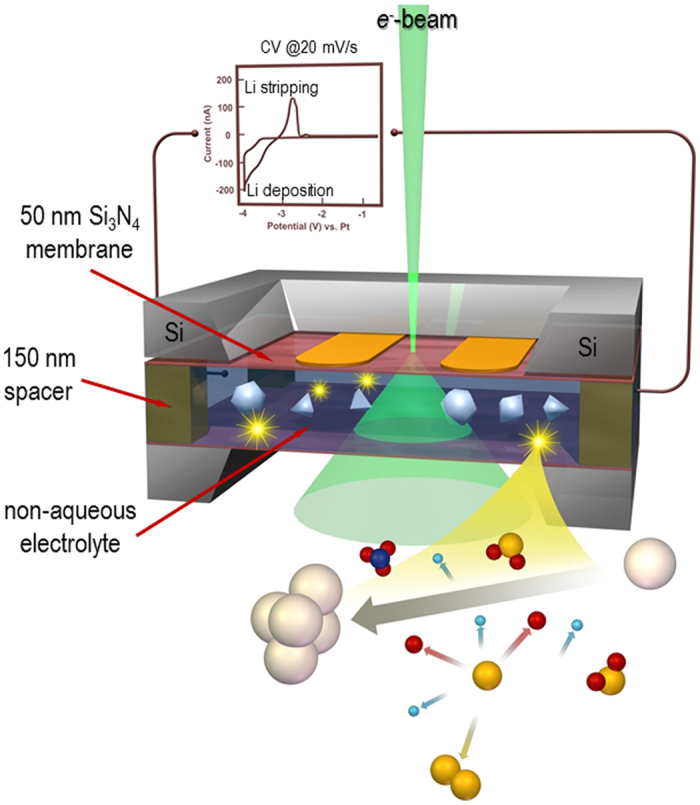
Illustration of an *in-situ* liquid electrochemical process in the scanning transmission (STEM) cell (*ec*-STEM) used for the Li dendrite deposition/stripping in the 1M LiPF_6_ in PC electrolyte with and without the H_2_O additive. The *in-situ* liquid *ec*-STEM cell is made from two silicon microchips containing 50 nm thick silicon nitride membranes transparent to the electron beam and three Pt microelectrodes aligned parallel to each other. The top electrochemical microchip has a 500 nm SU-8 spacer and the bottom microchip has a 150 nm gold spacer giving a nominal spacing of 650 nm. The electron beam passes through the electrolyte and two SiN_x_ membranes permitting the recording of the Li deposition and stripping in *real-time* at high spatial and temporal resolutions during the cyclic voltammetry or galvanostatic charge/discharge processes in STEM mode at a 2–3 μL min^−1^ flow rate and electron dose, which was kept below ≤0.3 electrons Å^−2^ s^−1^.

The measurements were performed with a commercially available Poseidon 510 (Protochips Inc., Raleigh, NC, USA) microfluidic *in-situ* electrochemical stage, which allows for the simultaneous observation of electrochemical measurements with video showing dynamics of Li dendrite deposition/stripping process at the Pt electrode surface in the liquid environment. The electrochemical cell itself is located in the tip of the TEM holder with two Si microchips (a top *ec*-chip with three Pt electrodes, a 500 nm SU-8 spacer and a 50 nm Si_3_N_4_ membrane and a bottom chip with a 150 nm spacer and a 50 nm Si_3_N_4_ membrane) sealed within the tip by two O-rings (Fig. 1). This configuration is then used to form an operating battery with an anode, cathode and non-aqueous liquid electrolyte. The microfluidic channels are integrated inside the holder to permit the circulation of the electrolyte at a 3 μL min^−1^ flow rate, thereby providing the Li^+^ ions for the battery operation. This approach permits the direct observation of the electrochemical processes at the anode/electrolyte interface without the need for a Li metal source. To prevent unwanted contamination of the battery electrolyte (1M LiPF_6_ in PC) by H_2_O and O_2_, the electrolyte solutions were prepared and the *operando* cell was assembled in an Ar glove box. The control experiment performed on an electrolyte without the additive had a residual H_2_O content of 10 ppm, while the electrolyte with the additive was designed to have an H_2_O content of 50 ppm^13^ (details on the measurement of H_2_O content are given in the Supplementary Information and shown in Fig. 1). The cyclic voltammetry experiments (CV) were conducted with a Gamry Reference 600 potentiostat scanning at a rate of 20 mV s^−1^, with a simultaneous recording of the video sequence of the Li deposition/stripping at the Pt electrode (also shown in Fig. 1). Full movies of the experiments are provided in the Supplementary Information as Supplementary Movies 1 and 2 (SM1 and SM2).

## Additional Information

**How to cite this article**: Mehdi, B. L. *et al*. The Impact of Li Grain Size on Coulombic Efficiency in Li Batteries. *Sci. Rep.* **6**, 34267; doi: 10.1038/srep34267 (2016).

## Supplementary Material

Supplementary Information

Supplementary Video 1

Supplementary Video 2

Supplementary Video 3

Supplementary Video 4

Supplementary Video 5

Supplementary Video 6

## Figures and Tables

**Figure 2:**
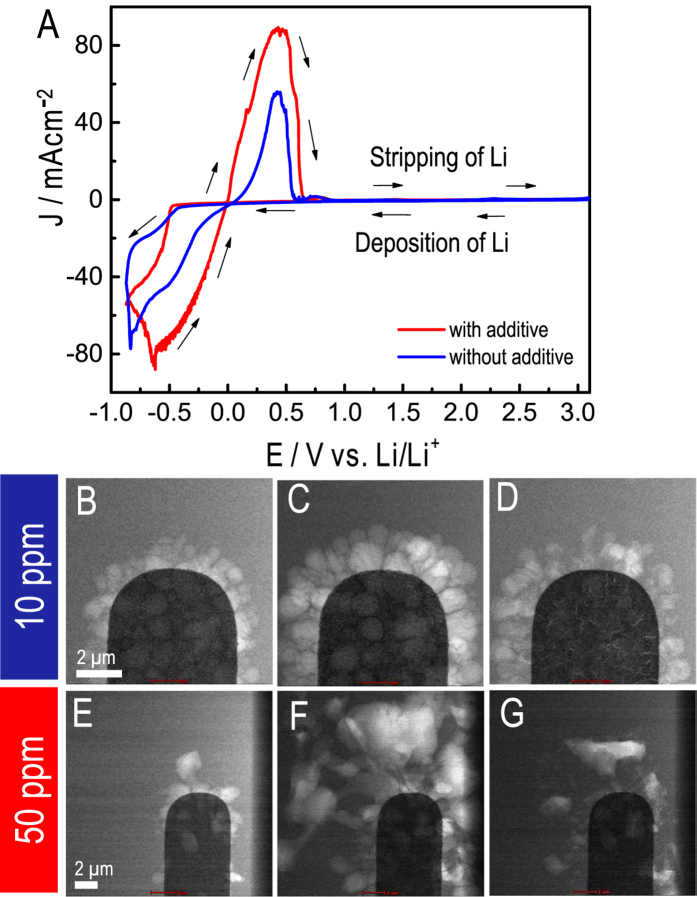
Direct observation of dynamic Li dendrite grain growth during the electrodeposition/stripping process in a Li-ion battery. (**A**) Cyclic voltammograms obtained from the *operando ec*-(S)TEM cell showing the charge-discharge cycle for the electrolytes with 10 and 50 ppm additions of H_2_O. Bright field (BF) images showing the deposition/stripping of Li for the electrolyte without additives (i.e., 14 ppm residual H_2_O) (**B**) at the start of the deposition, (**C**) at the peak of the deposition and (**D**) after the discharge is complete. BF images showing the same (**E–G**) for the electrolyte containing 50 ppm H_2_O.

**Figure 3:**
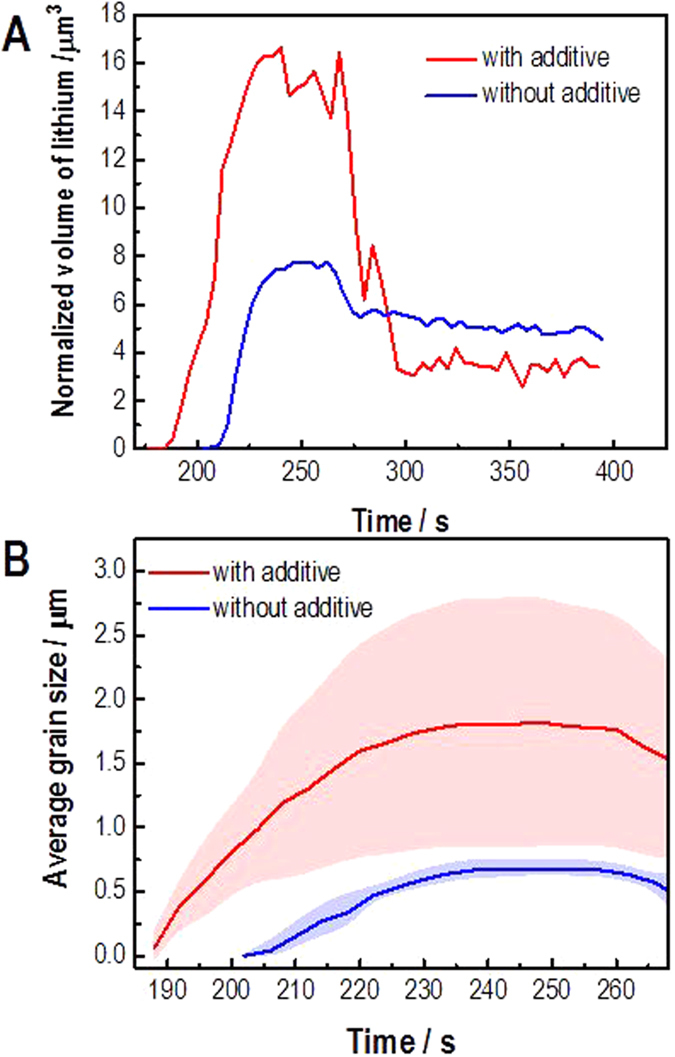
Experimental quantification of grain size in the Li dendrites. (**A**) Quantified mass of the Li deposited at the electrode-electrolyte interface measured by the automated tracking algorithm. Note that the Li deposition is initially increased for the electrolyte with the 50 ppm H_2_O additive, but the final amount of Li deposited after the cycle is complete is less, i.e., the cycling is more reversible for the electrolyte with the additive. (**B**) Average grain size for the charge/discharge cycle in (**A**) showing that the grain size of the deposits is larger for the electrolyte containing the additive.

**Figure 4:**
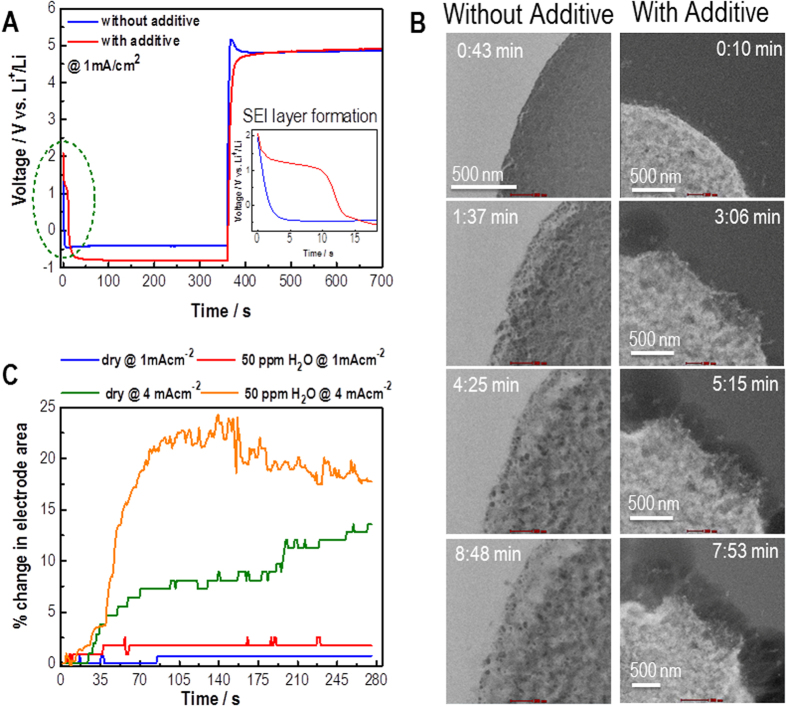
Voltage profile for charge-discharge cycling and dendrite formation. (**A**) Li deposition-stripping for a 1 mA cm^−2^ current density. Inset shows the distinct plateau for the SEI layer formation for the electrolyte with additive. (**B**) BF (left) and HAADF (right) STEM images of the interface between the Pt working electrode and the electrolyte during the Li deposition (charging process) at 4 mV cm^−2^. Images show the rapid expansion of the electrode surface in both electrolytes, the difference in diffusion of the Li^+^ cations along the grain boundaries of the Pt electrode and the formation of the Li grains with time. (**C**) Quantified % change in the Pt electrode area during charging at either a 1 or 4 mA cm^−2^ current density in the electrolytes with and without the additive.

**Figure 5:**
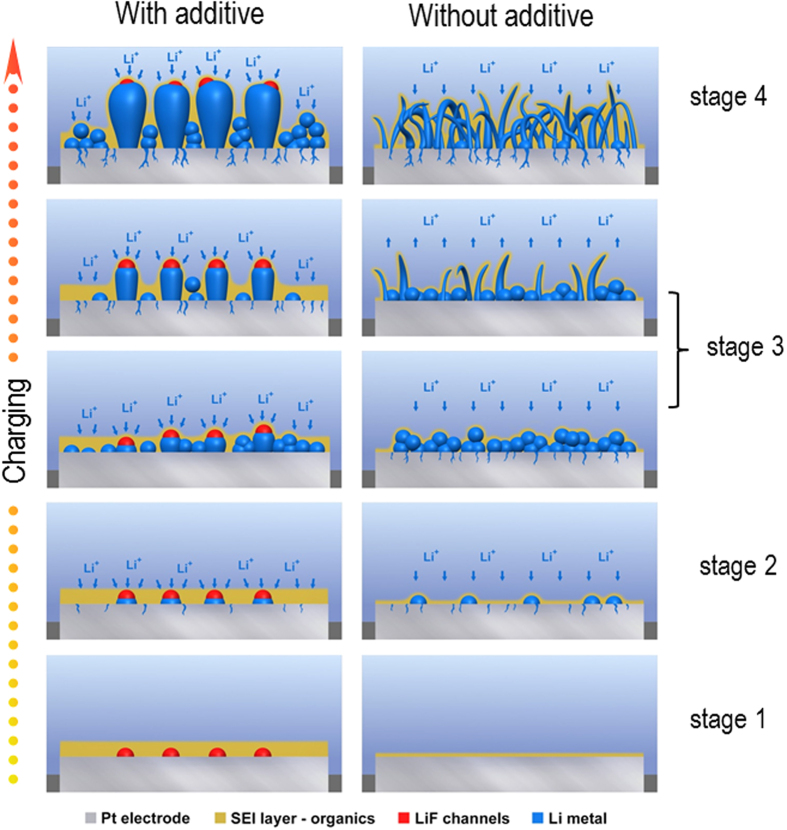
Schematic model illustrating the formation of two distinct forms of Li dendrite structures in the presence (left) and absence (right) of additives. In both cases, Stage 1 leads to the formation of the SEI layer. However, the electrolyte with the additive forms a layer containing a large amount of LiF, whereas without the additive the SEI layer is very thin. Stage 2 shows the initial nucleation stage of the Li nanoparticles leading to the full growth of the large Li grains (in the presence of the additive) and the long rods (without the additive) shown in Stage 3. Stage 4 of the Li deposition demonstrates the advanced growth and development of uniformly sized columnar grains (left) and a mossy Li deposit (right). The continued growth of the columnar grains is possible due to the significant amount of LiF present, which enables “fast Li^+^” diffusion through the LiF channels to the metal surface.
