# Supplementary material for: The Impact of Li Grain Size on Coulombic Efficiency in Li Batteries
Source: Sci Rep. 2016 Oct 5;6:34267. doi: 10.1038/srep34267 (PMC5050435; doi:10.1038/srep34267)
Supplement: Supplementary Information [file srep34267-s1.pdf]

The Impact of Li Grain Size on Coulombic Efficiency in Li Batteries

B. Layla Mehdi*[1,2], Andrew Stevens[3], Jiangfeng Qian[1,4], Chiwoo Park[5], Wu Xu[1,4], Wesley A. Henderson[4], Ji-Guang Zhang[1,4], Karl T. Mueller[1,6,7], and Nigel D. Browning*[1,2,8]

[1]Joint Center for Energy Storage Research (JCESR), Pacific Northwest National Laboratory (PNNL), Richland, WA 99352, USA

[2]Physical and Computational Science Directorate, PNNL, Richland, WA 99352, USA

[3]National Security Directorate, PNNL, Richland, WA 99352, USA

[4]Energy and Environmental Directorate, PNNL, Richland, WA 99352, USA

[5]Industrial and Manufacturing Engineering, Florida State University, Tallahassee, FL 32306, USA

[6]Environmental Molecular Sciences Laboratory, PNNL, Richland, WA 99352, USA

[7]Department of Chemistry, Penn State University, University Park, PA, 16802, USA

[8]Materials Science and Engineering, University of Washington, Seattle, WA 98195, USA

*E-mail: Layla.Mehdi@pnnl.gov and Nigel.Browning@pnnl.gov

Contents

1. **Supplementary movies showing *real-time* deposition/striping of Li dendrite under potentiostatic and galvanostatic conditions**

    a. **SM1:** Movie showing the deposition/stripping of Li dendrites without the additive (10 ppm of H_2O) with a 20 mV s^{-1} scan rate in a 1M $LiPF_6$ in PC electrolyte.

    b. **SM2:** Movie showing the deposition/stripping of Li dendrites with the additive (50 ppm of H_2O) with a 20 mV s^{-1} scan rate in a 1M $LiPF_6$ in PC electrolyte.

    c. **SM3:** Movie showing the deposition/stripping of Li dendrite without the additive (10 ppm of H_2O) with a 1 mA cm^{-2} current density.

    d. **SM4:** Movie showing the deposition/stripping of Li dendrite with the additive (50 ppm of H_2O) with a 1 mA cm^{-2} current density.

    e. **SM5:** Movie showing the deposition/stripping of Li dendrite without the additive (10 ppm of H_2O) with a 4 mA cm^{-2} current density.

f. **SM6:** Movie showing the deposition/stripping of Li dendrites with the additive (50 ppm of H_2O) with a 4 mA cm^{-2} current density.

2. **Additional data**

a. Supplementary Figure 1. Progress of the hydrolysis reaction in the 1M $LiPF_6$ in PC electrolyte after the addition of the H_2O additive.

b. Supplementary Figure 2. (A) BF STEM image of the frame extracted from the movie SM1 showing the Li growth at the Pt electrode surface from the 1M $LiPF_6$ in PC electrolyte without the additive (10 ppm of H_2O).

c. Supplementary Figure 3. (A) BF STEM image of the frame extracted from the movie SM2 showing the Li dendrite growth at the Pt electrode surface from the 1M $LiPF_6$ in PC electrolyte with the additive (50 ppm of H_2O).

d. Supplementary Figure 4. Voltage profile of the Li deposition/stripping processes with a 4 mA cm^{-2} current density in the 1M $LiPF_6$ in PC electrolyte.

1. **Additional data**

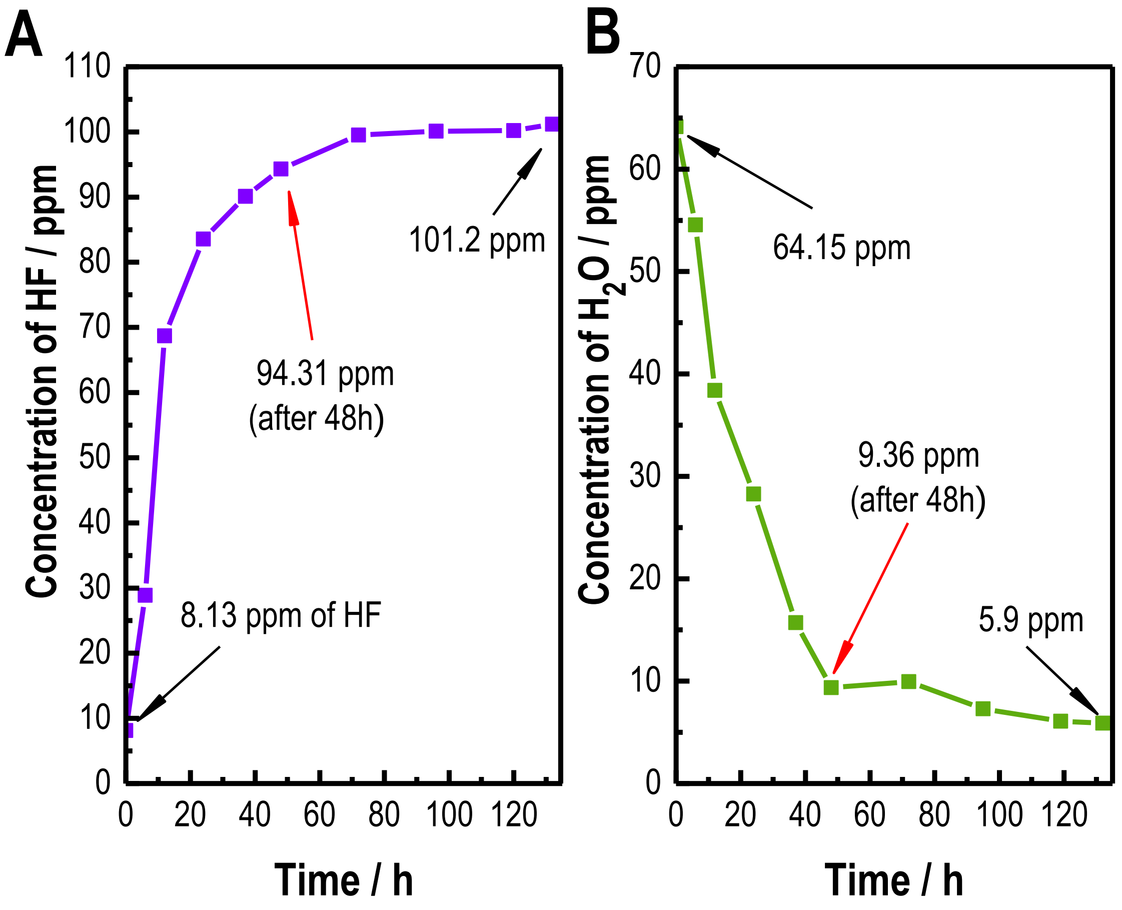

Supplementary Figure 1 | Progress of the hydrolysis reaction in the 1M $LiPF_6$ in PC electrolyte after the addition of the H_2O additive. The presence of the 50 ppm of H_2O changed the total concentration of the HF and H_2O in the $LiPF_6$-based electrolyte, leading to the initial linear increase in the HF concentration and corresponding decrease in the concentration of the H_2O, which was quickly consumed by the hydrolysis reaction. After aging the electrolyte for 48 h, the concentration of the HF reached a plateau at approximately 100 ppm and < 10 ppm of H_2O. The electrolyte used in the *in-situ* liquid *ec*-STEM experiments where therefore stored for 48 h prior to the experiments. Note that the initial concentration of water in the PC solvent was approximately 14 ppm.

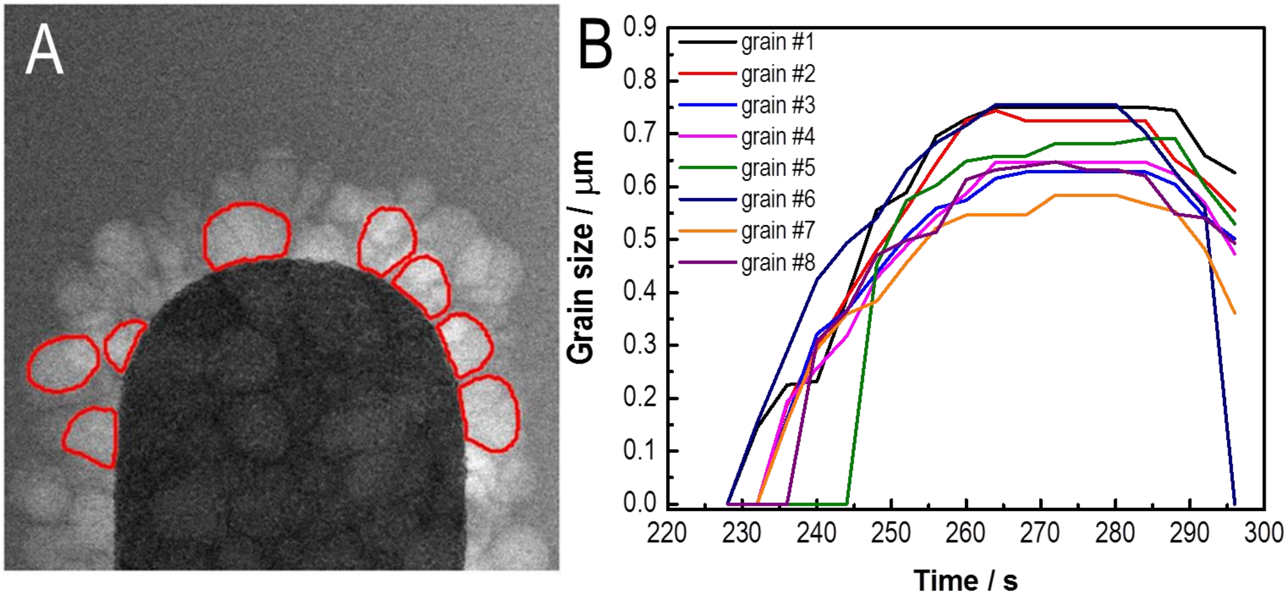

Supplementary Figure 2 | (A) BF STEM image of the frame extracted from the movie SM1 showing the Li growth at the Pt electrode surface from the 1M $LiPF_6$ in PC electrolyte without the additive (10 ppm of H_2O). The image shows the application of multitarget tracking algorithm used to extract the quantitative information about the size change with time of the eight individual grains during the Li dendrite deposition/stripping processes. (B) Quantitative size distribution of the eight individual Li grains identified in Supplementary Figure 5A and extracted from the movie SM1, showing the uniform Li grain growth with an average Li grain size distribution of 0.42 to 0.56 µm, in electrolyte without additive. The average grain size for the electrolyte without the additive was 0.48 µm as illustrated by the blue curve in Figure 2B.

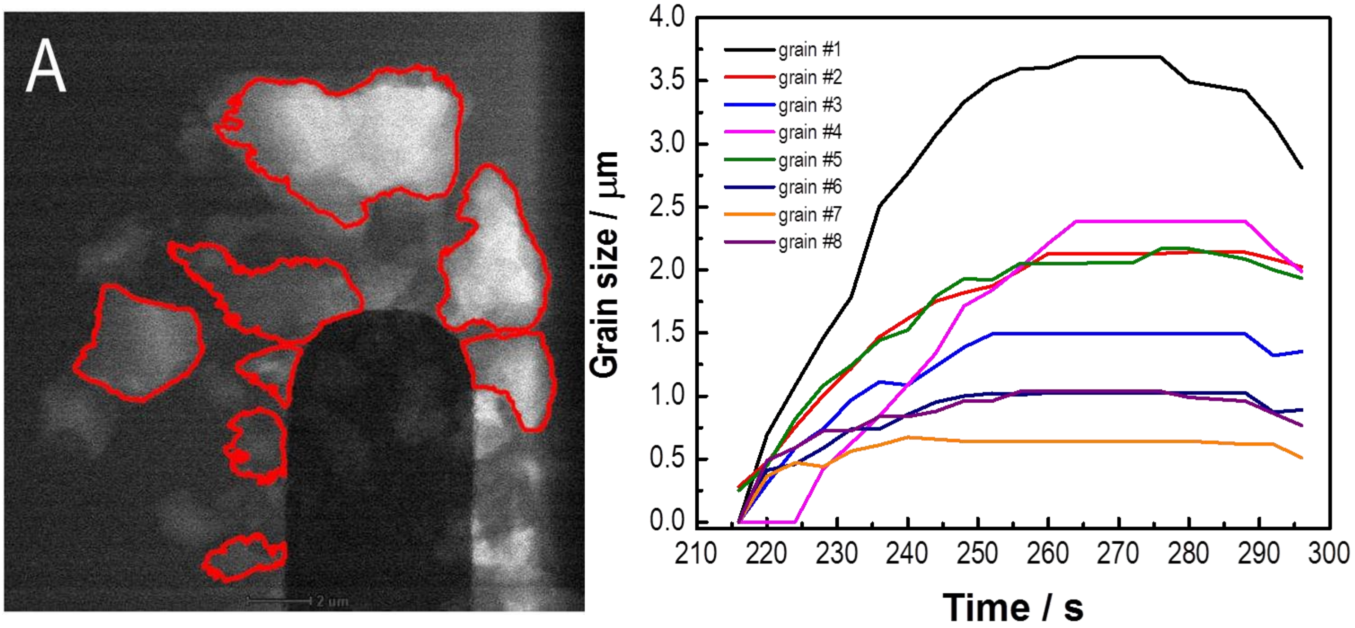

Supplementary Figure 3 | (A) BF STEM image of the frame extracted from the movie SM2 showing the Li dendrite growth at the Pt electrode surface from the 1M $LiPF_6$ in PC electrolyte with the additive (50 ppm of H_2O). The image shows the application of the multitarget tracking algorithm used to extract the quantitative information about the size change with time of the eight individual grains during the Li dendrite deposition/stripping process. (B) Quantitative size distribution of the eight individual Li grains identified in Supplementary Figure 5A and extracted from the movie SM2, showing the nonuniform Li grain growth with an average Li grain size distribution of 0.56 to 2.75 µm, in the electrolyte with the additive. The average grain size for the electrolyte with the additive was 1.3 µm as illustrated by the red curve in Figure 2B.

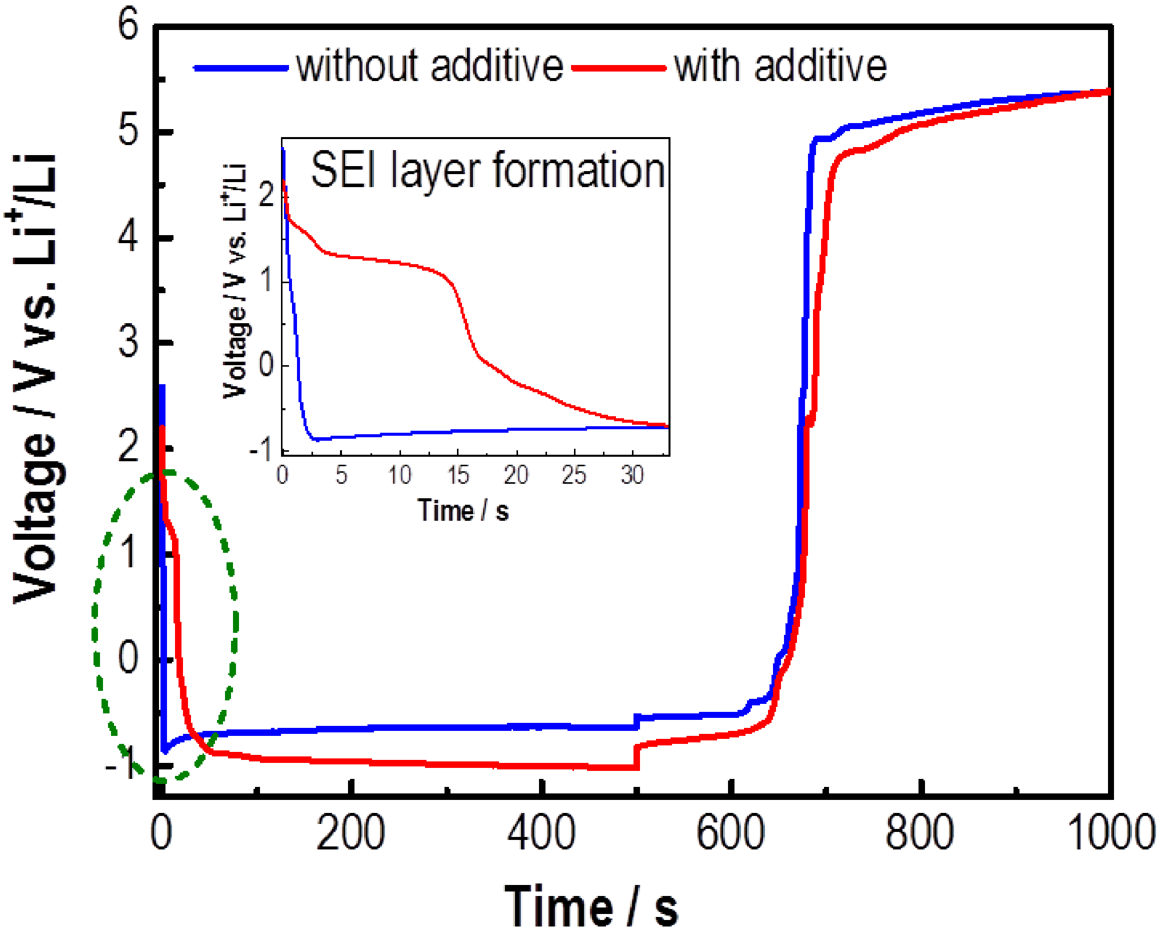

Supplementary Figure 4 | Voltage profile of the Li deposition/stripping processes with a 4 mA cm^{-2} current density in the 1M $LiPF_6$ in PC electrolyte. The inset shows the plateau for the SEI layer formation. The full dynamics of the Li deposition/stripping process is shown in Supplementary Movies 5 and 6. The electrolyte with the additive was aged for 48 h prior to the experiment.
